# Ion Channels in Endometrial Cancer

**DOI:** 10.3390/cancers14194733

**Published:** 2022-09-28

**Authors:** Bruna Pasqualotto Costa, Fernanda Bordignon Nunes, Francini Corrêa Noal, Gisele Branchini

**Affiliations:** 1 Laboratory fo Cellular, Molecular and Computational Biophysics, Universidade Federal de Ciências da Saúde de Porto Alegre (UFCSPA), Porto Alegre 90050-170, RS, Brazil; 2 Graduate Program in Pathology, Universidade Federal de Ciências da Saúde de Porto Alegre (UFCSPA), Porto Alegre 90050-170, RS, Brazil

**Keywords:** endometrial cancer, ion channels, tumor growth, tumor progression

## Abstract

**Simple Summary:**

Uterine or endometrial cancer is one of the most common types of cancer among the female population. Different alterations of molecules are related to many types of cancer. Some molecules called ion channels have been described as involved in the development of cancer, including endometrial cancer. We review the scientific evidence about the involvement of the ion channels in endometrial cancer and how some treatments can be developed with these molecules as a target. Even though they are involved in the progression of endometrial cancer, since they are present throughout the whole body, some possible treatments based on these could be studied.

**Abstract:**

Uterine or endometrial cancer (EC) is the sixth most common neoplasia among women worldwide. Cancer can originate from a myriad of causes, and increasing evidence suggests that ion channels (IC) play an important role in the process of carcinogenesis, taking part in many pathways such as self-sufficiency in growth signals, proliferation, evasion of programmed cell death (apoptosis), angiogenesis, cell differentiation, migration, adhesion, and metastasis. Hormones and growth factors are well-known to be involved in the development and/or progression of many cancers and can also regulate some ion channels and pumps. Since the endometrium is responsive and regulated by these factors, the ICs could make an important contribution to the development and progression of endometrial cancer. In this review, we explore what is beyond (ion) flow regulation by investigating the role of the main families of ICs in EC, including as possible targets for EC treatment.

## 1. Introduction

Cancer is considered the main public health problem and the second leading cause of global mortality [1]. In 2020, there were estimated to be more than 19 million new cases (including non-melanoma skin cancer) and 10 million deaths from cancer. In the female population, gynecological cancers are common, and uterine cancer is the sixth most common cancer among women worldwide. Approximately 417,000 new cases and 97,000 deaths caused by uterine cancer were diagnosed in 2020 [2].

Cell proliferation, migration, apoptosis, and differentiation are involved in cancer initiation and progression, and it is well-recognized that ion channels and transporters have a central role in regulating these processes.

For this review, a vast search was performed in the principal sources of biomedical literature to find studies involving the function and/or expression of ion channels in endometrial cancer. To give an overview of the topic, we also searched for complementary information about the general characteristics of each ion channel family. To perform the search about the role of IC in EC, the MeSH terms (((“Membrane Transport Proteins”[Mesh]) AND “Ion Channels”[Mesh]) AND “Endometrial Neoplasms”[Mesh]) were selected. In-cited literature was used as a search source to find and include other papers not shown in the initial search.

### 1.1. Endometrial Cancer

Most uterine cancers are usually referred to as endometrial cancer (EC), originating from the inner lining of the uterine cavity (endometrium) [3]. Based on the histological characteristics, stages, and hormone receptor expression, EC is classified into two types: endometrioid endometrial carcinoma (EEC; Type 1) and non-EEC subtype (NEEC; Type 2). EEC corresponds to more than 80% of the cases and is generally estrogen-dependent, and NEEC develops independently of estrogen [3]. The stages of endometrial cancer vary from I to IV according to the International Federation of Gynecology and Obstetrics (FIGO) [4] and the American Joint Committee on Cancer TNM staging system [5]. Higher stages correspond to a higher grade and have higher chances of cancer spreading throughout the body [6].

Carcinogenesis includes the support of proliferative signaling, the avoidance of growth suppressors, the resistance to cell death allowing for replicative immortality, and metastasis. The presence of genomic instability and mutations, inflammation, and reprogramming of energy metabolism are considered emerging hallmarks and enabling characteristics [7]. The abnormal proliferation of the endometrial glands increases in the gland/stroma ratio when compared to the endometrium of the proliferative phase of the cycle. The majority of endometrioid neoplastic lesions appear to evolve from endometrial hyperplasia (EH) without atypia to hyperplasia with atypia lesions (AEH) until well-differentiated EC [8,9]. It is believed that most ECs occur because of stimulation of the endometrium by unopposed estrogens. Endogenous or exogenous estrogen is not balanced simultaneously by progestogen, increasing the risk of inducing mitotic activity of the endometrial cells [10].

The endometrium can receive “unopposed” estrogenic stimulation by several routes or mechanisms: (I) iatrogenic (for example, hormone replacement with estrogens only); (II) production of estrogens by functional tumors (for example, granulosa cell tumor); (III) perimenopause, which leads to high levels of follicle-stimulating hormone (FSH), a decreased ovarian reserve, and frequent anovulatory cycles; (IV) obesity, which leads to insulin resistance, increased insulin levels, decreased levels of sex hormone-binding globulin (SHBG), and aromatization of androgens into estrogens; (V) polycystic ovary syndrome, which develops with hyperinsulinemia, an increased luteinizing hormone (LH)/FSH ratio, hyperandrogenemia, and anovulatory cycles [9]. Conversely, NEECs (Type 2) tend to be estrogen-independent, often associated with endometrial atrophy in postmenopausal women rather than with EH as in EEC (Type 1). Type 2 ECs are clinically more aggressive and are linked with a poorer clinical prognosis [11].

The main known risk factors for developing endometrial cancer are metabolic syndrome, use of oral contraceptives, and null parity [12,13,14]. The incidence of EC is steadily increasing, mainly as a result of raised rates of obesity and population aging [15]. Surgery (hysterectomy with bilateral salpingo-oophorectomy) is still the most frequent treatment for EC used in clinical practice [3]. Radiation and chemotherapy as adjuvant treatments may be recommended depending on the tumor degree differentiation. For those patients with metastatic disease or who wish to preserve their fertility, hormonal therapy (aromatase inhibitors, progestins, and LH-releasing hormone agonists) is an alternative treatment [16].

A favorable prognosis is found in most patients newly diagnosed with EC, with five-year relative survival rates of 81% [17]. However, it is expected that 15 to 20% of patients diagnosed early have recurrences or metastases after surgery [18]. It is estimated that radiotherapy and/or chemotherapy after surgical treatment benefit only 10 to 15% of patients [19]. Patients with recurrent EC or who have metastases have a poor prognosis, with survival rates of less than one year [20]. The success rate of hormonal treatments is also limited to the individual response profile as well as the positive expression of estrogen receptors by tumor cells [21].

Therapeutic resistance leads to highly harmful side effects for patients as it limits the use of medications and the way they are administered [22]. The identification of new biomarkers and therapeutic targets for EC is essential to broaden therapeutic approaches and increase the overall survival of patients. Increasing evidence points to ion transport mechanisms as an adjuvant in the carcinogenesis process, also offering novel therapeutic possibilities [23].

### 1.2. Ion Channels

Physiological processes such as the pH balance, volume, and cell cycle regulation, immune responses, secretion, muscle contraction, and electrical signals (nerves, muscles, and synapses) are mediated by the movement of ions between intracellular and extracellular fluid [24]. Ion channels and transporters (ICTs) are transmembrane proteins that strictly control the movement of ions across the cell membrane while maintaining the ionic gradients of cells. In this way, ICT assists in the selectively permeable nature of the cell membrane, functioning as gateways for charged ions that cannot diffuse freely through the lipid membrane barriers [25].

Ion channels are fast translocators and have pores that allow specific ions to cross the membrane in favor of an electrochemical gradient. Some channels, such as those dependent on electrical voltage, can detect the electrical potential opening or closing in response to the magnitude of the membrane potential. Ion channels can also be controlled by extracellular (neurotransmitter) and intracellular (second messenger) chemical signals or they can respond to mechanical and thermal stimuli. In contrast, transporters (also called ion pumps and exchangers) slow translocations, and the movement of ions occurs actively against the concentration gradient using energy, typically in the form of adenosine triphosphate (ATP) [24,25].

Rising evidence suggests that ion channels also play an important role in the process of carcinogenesis. Although cancer is not cataloged as a channelopathy, channels and ion pumps contribute to the progression of cancer by playing important roles in self-sufficiency in growth signals, proliferation, evasion of programmed cell death (apoptosis), angiogenesis, cell differentiation, migration, adhesion, and metastasis. [26,27,28]. Growth factors and hormones are well-known to be involved in the development and/or progression of many cancers and also can regulate some ion channels and pumps, contributing to carcinogenesis [29].

Given they are critically important in carcinogenesis, ICs may represent promising therapeutic targets, potentially combined with chemotherapy, immunotherapy, or other molecules that affect essential processes in tumor cells, such as oxidative stress and metabolic pathways [30]. The role of ion channels and transporters as potential therapeutic targets is one of the most innovative approaches to anticancer treatment [30]. However, the oncologic therapeutic strategy must be specific to a given ionic channel or pump to damage the targeted cell without causing toxic effects in other tissues expressing the same channels [27,29].

Channels are usually classified by the ion to which they are selective. They can be subdivided according to functional properties, such as a mechanism of regulation (Ca^+2^-activated) or a biophysical characteristic (inward rectifier). However, the receptor-operated channels (selective for either cations or anions) and novel transient receptor potential (TRP) channels (which discriminate poorly between monovalent and divalent cations) do not adhere to these simple rules [31].

#### 1.2.1. Potassium Channels

The potassium (K^+^) channels are a complex family of ion channels. They can be divided into four classes: (I) voltage-gated potassium channels (VGKC), (II) calcium-activated potassium channels (KCa), (III) inward rectifying potassium channels (Kir), and (IV) two-pore domain potassium channels (K2P) [26]. The K^+^ levels play an important role in membrane potential control, determination, and duration of the action potential, modulation of hormones’ secretion, and balancing excitatory signals in cells [32].

Changes in the cell cycle in tumor cells have been related to loss of function or altered expression of K^+^ channels in several tumor types [26]. At least in the case of VGKC and KCa, the control of cancer cell proliferation can occur through the modulation of membrane potential (V_m_), which, in turn, regulates transmembrane calcium (Ca^+2^) flow. Intracellular Ca^+2^ levels participate in cell cycle checkpoints’ control in normal and neoplastic proliferation [33].

#### 1.2.2. Sodium Channels

There are two very different types of sodium (Na^+^) channels: (I) voltage-gated sodium channels (VGSC) and (II) epithelial sodium channels (ENaC). Present in absorptive epithelia (such as distal twisted tubules of the kidneys, colon, lungs, and ducts of the salivary gland), the ENaC are involved in Na^+^ absorption and a play key role in maintaining Na^+^ homeostasis, which is linked directly to the volume of extracellular fluid [34]. However, VGSC are involved in the initial phase of the action potential in most cells, being important for the generation and propagation of the action potential [26].

VGSC are formed mainly by a pore-forming multi-spanning integral membrane glycoprotein (α subunit) that can be associated with one or more regulatory β subunits. The β subunits are single-span integral membrane proteins that modulate the sodium current. They can also act as cell adhesion molecules in terms of interaction with extracellular matrix molecules, communication between adjacent cells, regulation of cell migration, cellular aggregation, and interaction with the cytoskeleton [35]. Aberrant expression/function of VGSCs is related to cell migration, invasion, and tumor metastasis [26].

#### 1.2.3. Chloride Channels

Chloride (Cl^−^) is the most abundant anion in the extra- and intracellular spaces. Cl^−^ transport through the plasma membrane is involved in numerous physiological processes, from homeostasis to volume control and regulation of excitable cells [36]. The chloride channels (ClC) are a family of anion channels that mediate the transport of Cl^−^ ions across the cell. They can act through voltage dependency, triggered by calcium, or activated by several ligands and second messengers, and can be divided into two major classes: voltage-dependent Cl^−^ channels of the ClC family, and the cystic fibrosis transmembrane conductance regulator (CFTR) [37].

Dysregulation of Cl^−^ channels has been reported in multiple cancer types related to cell migration, invasion, and metastasis [26,27]. The Cl^−^ intracellular ion channels (CLICs) are an emerging class involved in cancer development [38]. CFTR is expressed in the epithelial cells of various tissues and organs. Although defective CFTR leads to cystic fibrosis, dysregulation of CFTR can promote or suppress cancer progression [39].

#### 1.2.4. Calcium Channels

Calcium is an important signaling molecule and serves as a second messenger for several fundamental cellular processes such as cell cycle control, migration, and apoptosis [40]. Regulation of intracellular Ca^+2^ levels involves the flow of Ca^+2^ through the plasmatic membrane and the release of intracellular Ca^+2^ stocks in the endoplasmic reticulum and mitochondria [26].

The Ca^+2^ channels are generally activated in response to membrane depolarization and mediate the influx of calcium in response to action potentials and depolarizing signals [41]. Calcium channels can be classified according to their activation mechanism: (I) voltage-gated calcium channels (VGCCs), (II) receptor-operated calcium channels (ROCCs), (III) store-operated calcium channels (SOCCs), (IV) transient receiver potential channels (TRPs), (V) acid-sensing ion channels (ASICs), and (VI) stretch-activated ion channels (SAICs) [42]. These channels play important roles in human physiology and it is not a surprise that calcium channel disorders are associated with tumor cell growth, survival, angiogenesis, and migration [26,42,43].

#### 1.2.5. Porins

Voltage-dependent anion channel (VDAC), also known as a mitochondrial porin, regulates metabolites exchange between cytosol and mitochondria, cellular energy homeostasis, and is involved in mitochondria-mediated apoptosis. There are three VDAC isoforms (VDAC1, VDAC2, and VDAC3), and alterations in VDAC expression have been reported already in human pathologies, including cancer [44].

Aquaporins (AQP) are integral membrane proteins that serve as channels and enable the regulated transport of water essential to homeostasis in response to osmotic gradients created by the active transport of solutes. AQP isoforms identified in mammals present in multiple organs and tissues are involved in many biological functions. Altered AQP expression is related to carcinogenesis in diverse tissues, especially motility, invasiveness, and angiogenesis [45,46].

## 2. ICs’ Expression in Endometrial Cancer

Ion channels play an important regulatory role in receptivity and embryo implantation in the endometrium. Abnormalities in ion transport are related to endometrial diseases such as infertility and cancer [47]. The roles of ion channels in cancer-related cellular behaviors and the specific expression and functional profiles of various channels characteristic of certain human cancers have been studied as potential diagnostic and therapeutic targets [28]. Table 1 shows evidence collected to date about the role of ICT in endometrial cancer (Table 1).

### 2.1. Potassium Channels

The human EAG-related gene (hERG), which encodes the alpha subunit of the Kv11.1 channel, belongs to the EAG (ether-à-go-go) family, a subfamily of the Kv channels encoded by the KCNH gene family [48]. Aberrant hERG expression in various cancer cells has been correlated with cancer progression [49]. Cherubini et al. (2000) analyzed human samples and found high gene and protein hERG expression in endometrial cancers compared to normal and hyperplastic endometrium. The authors suggested the possible use of hERG K^+^ channels’ expression as a discriminatory molecular marker between cancerous and non-cancerous endometrium [50]. Suzuki et al. (2004) evaluated the multiple pore-forming and regulatory subunits of voltage-gated potassium (Kv) channel gene expression in uterine cancer cells. Although they did not use non-tumor endometrial cells, hERG-KCNE channel complexes may be selectively involved in the proliferation of endometrial cancer cells. However, the hERG channel blocker E-4031 did not reduce endometrial cancer cell proliferation [51].

hERG plays a role in depolarizing and hyperpolarizing the membrane potential. K^+^ channel-dependent hyperpolarization seems critical to cell cycle progression (G1 to S phases). Ca^+2^ influx evoked by hyperpolarization and the opening of more KCa is associated with mitogenic factors’ synthesis [52]. KCa are divided into three subfamilies: big conductance (BKCa; activated by depolarization and/or by increases in intracellular [Ca^+2^]), intermediate conductance (IKCa; activated by low intracellular [Ca^+2^]), and small conductance (SKCa; activated by low intracellular [Ca^+2^]) [53].

**Table 1:** Expression of ICTs in endometrial cancer.

Ion Channel or Transporter	Cellular Process(es) or Pathway(s)	Methods of Analysis	Type of Alteration	Reference
**Potassium Channels**				
Kv11.1 alpha subunit (hERG)	Differentiation and growth	Endometrial samples: RT-PCR and IHC	▲frequency of hERG gene and protein expression in EC compared to NE	[50]
Kv11.1 alpha subunit (hERG)	Differentiation and growth	In vitro: RT-PCR and specific K+ channel blockers	(+) expression of hERG channel, and their potential auxiliary KCNE subunits are involved in cell proliferation ▼hERG did not reduce cell proliferation	[51]
IKCa1	Tumor progression	Endometrial samples: RT-PCR and WB In vitro: Downregulation and activity inhibition of IKCa1 In vivo: Mouse model of EC	▲gene and protein expression of IKCa1 in EC specimens compared to NE ▼IKCa1 suppressed cell proliferation and restrained cancer growth	[54]
KCa3.1	Cell proliferation, migration, and invasion	In vitro: Downregulation and activity inhibition of KCa3.1	▼KCa3.1 channel inhibits cell proliferation, cell cycle progression, migration, and cellular invasion	[55]
BKCa	Cancer initiation and development	Endometrial samples: IHC In vitro: Downregulation of BKCa	▲ BKCa expression in EC tissues compared to NE ▼ BKCa inhibited cell proliferation and migration	[56]
BKCa	Cell proliferation and migration	In vitro: Overexpression and downregulation of BKCa In vivo: Mouse xenograft model	▲ BKCa stimulated proliferation and migration ▼ BKCa inhibited cell proliferation and migration and impaired tumor growth in vivo	[57]
K2P	Cell proliferation	Endometrial samples: RT-PCR and IHC In vitro: K2P activity inhibition	▲ TREK-1 expression in proliferative phase of endometrium ▼ Cell proliferation by K2P channel blockers	[58]
Calcium channels				
Cav1.3	Cell proliferation and migration	Endometrial samples: IHC In vitro: Downregulation of Cav1.2 channel and E2 treatment	▲ expression of Cav1.3 in EC and AEH specimens compared to NE ▼ Cav1.3 inhibited cell proliferation and migration	[59]
Cav1.3	Cell proliferation, apoptosis, and autophagy	In vitro: Cav1.3-antagonist	▼ Cav1.3 suppressed cell proliferation and migration ▼ Cav1.3 increased apoptosis and autophagy	[60]
CACNA2D3	Cell proliferation and migration	Endometrial samples: RT-PCR and IHC In vitro: Overexpression of CACNA2D3 and P4 treatment In vivo: Mouse xenograft model	▼ expression of CACNA2D3 in EC tissues and cells ▲ CACNA2D3 inhibited cell proliferation and migration ▲ CACNA2D3 suppressed tumor growth in vivo	[61]
TRPM4	Cell proliferation and migration	In silico: Bioinformatics analysis In vitro: Downregulation of TRPM4 channel and E2 treatment	▼ TRPM4 expression levels correlated with poor clinical outcomes and EC cell proliferation ▼ TRPM4 promoted proliferation and migration	[62]
TRP	Mobility and invasiveness	Endometrial samples: RT-PCR In vitro: Primary endometrial stromal and epithelial cell culture	▲TRPV2 and TRPC1 expression in EC is associated with high-risk cancer and high EMT status ▲TRPM4 mRNA expression was related to lower-risk EC and low EMT status	[63]
TRPV4	Cell proliferation and metastasis	In silico: Proteomic and bioinformatics analysis In vitro: Downregulation and overexpression of TRPV4 In vivo: Mouse xenograft model	▼ TRPV4 decreased Ca^+2^ influx and metastatic ability ▼ TRPV4 reduced peritoneal nodules in vivo ▲ TRPV4 showed the opposite effects in vitro and in vivo models	[64]
Chloride channels				
CFTR	Cell proliferation and migration	Endometrial samples: RT-PCR and IHC In vitro: Downregulation of CFTR	▲ CFTR expression in EC compared to NE ▼ CFTR increases proliferation and migration	[65]
Sodium channels				
Na_v_1.7	Tumor progression	Endometrial samples: RT-PCR In vitro: Primary EC cell culture and inhibition of Na_v_ 1.7	▲ Nav1.7 expression in EC tissues ▲ Nav1.7 associated with poor prognosis ▼ Nav1.7 induced apoptosis and reduced the invasiveness ability	[66]
Porins				
AQP1	Angiogenesis	Endometrial samples: IHC	(+) AQP1 expression in small vessels and microvessels ▲ AQP1 expression in EC compared to NE ▲ AQP1 correlated with tumor angiogenesis and poor prognosis	[67]
AQP2	Cell migration, invasion, and adhesion	Endometrial samples: IHC and WB In vitro: Downregulation of AQP2	▲ AQP2 expression in EC compared to NE ▼ AQP2 attenuated migration, invasion, and adhesion, but not proliferation	[68]
AQP5	Cell migration	In vitro: Downregulation of AQP5	▼AQP5 attenuated cell migration	[69]
AQP3	Cancer cell differentiation	Endometrial samples: IHC	AQP3 expression is correlated with EC at an earlier stage and lower histological grade	[70]
VDAC	Tumor progression	Endometrial samples: RT-PCR and WB	▲ VCAC1 and VDAC3 expression in EC compared to NE VCAC1 and VDAC3 expression correlates with tumor progression	[71]

▲ increase; ▼ decrease; (+) positive. RT-PCR (reverse transcription-polymerase chain reaction); IHC (immunohistochemistry); WB (Western blot); NE (normal endometrium); E2 (estrogen); AEH (atypical endometrial hyperplasia).

Wang et al. (2007) demonstrated higher mRNA and protein expression of IKCa1 in endometrial cancer specimens than in normal endometrium and atypical hyperplasia specimens. The pharmacological inhibition of IKCa1 (clotrimazole and TRAM-34) and the downregulation by siRNA against IKCa1 suppressed the EC cell proliferation and arrested the cell cycle. Nude mice treated with clotrimazole and TRAM-34 showed restrained endometrial cancer growth, suggesting that IKCa1 channels may be a new target for the treatment of EC [54]. Similarly, Zhang et al. (2015) evaluated the role of the intermediate-conductance KCa3.1 channel in HEC-1-A and Ishikawa endometrial cancer cells. The gene silencing and pharmacological blockage of the KCa3.1 suppressed cell proliferation and cell cycle progression, and decreased the expression of cyclin D1 and MMP-2, proteins involved in tumor migration and invasion [55].

Wang et al. (2018) revealed higher expression of BKCa in endometrial adenocarcinoma tissues compared to normal endometrium and atypical endometrial hyperplasia. Furthermore, in vitro assays showed that RNAi-mediated knockdown of BKCa inhibited endometrial cancer cell (Ishikawa) growth, possibly via inactivation of the MEK/ERK pathway [56]. On the other hand, overexpression of BKCa promoted proliferation and migration of endometrial cancer HEC-1-B cells. BKCa knockdown decreased these pro-carcinogenic effects and suppressed the growth of the HEC-1-B xenografts in nude mice. The treatment with the selective BKCa channel inhibitor Iberiotoxin (IbTX) decreased HEC-1-B cell proliferation and migration [57]. 

K2P is a ‘‘leak channel’’ essential for maintaining a negative resting membrane potential [72]. TWIK-related K^+^ (TREK) channels, a subgroup of K2P channels, have been related to endometrial cancer. According to Patel et al. (2013), the proliferative endometrium expresses higher TREK-1 levels compared to the secretory endometrium, possibly linked to increased cell division in this phase of the menstrual cycle. The K2P channel blockers (methanandamide, lidocaine, zinc, and curcumin) showed antiproliferative effects in endometrial cancer in vitro [58]. K2P channels are expressed in a variety of human cell types. Aberrant expression and function are related to human diseases, such as cancer, and therapeutic regulation of K2P channel activity has been studied in different pathologies [73].

### 2.2. Calcium Channels

Different subunits of VGCCs demonstrated some degree of participation in cancer progression and development [74]. The L-type calcium channel α 1D subunit (Cav1.3) belongs to the family of VGCC channels. Immunohistochemical results showed high Cav1.3 expression in endometrial carcinoma and atypical endometrial hyperplasia tissues compared to benign endometrial tissues [59]. Sex steroid hormones, including estrogens, can modulate the expression of ion channels in cancer cells, especially in hormone-sensitive tissues [75]. According to Hao et al. (2015), shRNA-mediated Cav1.3 silencing suppressed endometrial cancer cell proliferation and migration. Although E2 treatment increased cell migration, its effect was partly inhibited by Cav1.3 deletion in EC cells. Bao et al. (2012) demonstrated the Cav1.3-antagonist nifedipine significantly suppressed endometrial carcinoma Hec-1A cells’ proliferation and migration in vitro. However, beyond apoptosis, autophagy was also induced in Hec-1A cells by nifedipine as a mechanism of cell survival. Autophagy inhibitor 3-MA enhanced nifedipine-induced cell death [60].

A recent work evaluated the role of CACNA2D3 (calcium voltage-gated channel auxiliary subunit α2δ3) in endometrial cancer. Kong et al. (2020) reported low expression of CACNA2D3 in endometrial cancer tissues and endometrial cell lines (Ishikawa and RL95-2) compared to adjacent healthy endometrial tissues. Unlike the other channels, overexpression of CACNA2D3 decreased cell proliferation and migration, and increased apoptosis and Ca^+2^ influx in EC cells. Overexpression of CACNA2D3 also decreases tumor growth in a mouse xenograft model. Progesterone (P4) signaling seemed to act in the upregulation of CACNA2D3 expression (in vivo and in vitro) since CACNA2D3 knockdown blocked the function of P4 [61].

Also studied in EC are TRP channels, which are Ca^+2^-permeable ion channels. Li et al. (2020) evaluated endometrial cancer calcium-activated TRPM4 channel gene expression data through The Cancer Genome Atlas (TCGA) datasets. Low TRPM4 expression levels were correlated with poor clinical outcomes and survival. The TRPM4 silencing in endometrial cancer AN3CA cells promoted proliferation and migration [62]. Recently, Eynde et al. (2022) investigated the TRP channel mRNA expression patterns in malignant endometrial tissues and tumor microenvironment epithelial and mesenchymal cells. The study cross-referenced TRP channel expression data with the epithelial to mesenchymal transition (EMT) status, a change that allows cells to acquire mobility and invasiveness. Calcium-permeable TRPV2 and canonical TRPC1 channels’ expression in both endometrial cancer biopsies and cancer cells were associated with high-risk biopsies and a high EMT status. In contrast, TRPM4 mRNA expression was higher in low-risk cancer tissues and cancer cells and with lower EMT status [63].

Li et al. (2020) also demonstrated that high expression of TRPV4 (transient receptor potential vanilloid 4) is associated with EC progression in vitro and in vivo. TRPV4 depletion (shTRPV4) decreased the calcium influx and metastatic ability in Ishikawa cells, and TRPV4-overexpression (OETRPV4) increased calcium levels and metastatic ability in HEC-1A cells. In vivo tumor xenograft models allowed for an evaluation of the number of metastatic peritoneal nodules. The xenograft model with Ishikawa cells (higher TRPV4 expression) showed a reduction in peritoneal nodules, while xenograft model HEC-1A cells (lower TRPV4 expression) increased the peritoneal nodules. Treatment with a TRPV2 agonist (GSK1016790A) and antagonist (HC067047) reverted the results. The authors also proposed that TRPV4 and Ca^+2^ could promote metastasis by regulating the cytoskeleton through the RhoA/ROCK1 pathway [64].

### 2.3. Chloride and Sodium Channels

Although less studied in endometrial cancer, Cl^−^ and Na^+^ channels have been demonstrated to be involved in cancer progression. According to Xia et al. (2017), CFTR chloride channel expression is upregulated in endometrial carcinoma tissue compared to non-tumoral tissues. However, the specificity inhibitor CFTR(inh)-172 intensified the proliferative and migrative capability of endometrial Ishikawa cells in vitro [65]. Although not directly studied in endometrial cancer, overexpression of chloride channel-3 (CLC-3) was associated with migration and invasion in ectopic endometrial cells from patients with endometriosis [76] and progression of human cervical carcinoma [77].

Voltage-gated sodium channel Nav1.7 was highly expressed in endometrial carcinoma compared to adjacent non-tumoral tissue. Results from Liu et al. (2019) associated Nav1.7 levels with the tumor size, local lymph node metastasis, and patient survival. In vitro experiments with Nav1.7 blocker (PF-05089771) induced cancer cell apoptosis and reduced the invasion ability of isolated cells from EC biopsies [66].

### 2.4. Porins

Accumulating evidence has been suggesting that aquaporins are involved in the tumorigenesis process [45]. Aquaporin-1 (AQP1) was widely expressed in most secretory and absorptive epithelia and in the endothelial cells of microvessels. An imbalance in AQP1 could indicate a possible involvement in tumor angiogenesis and cell proliferation [78]. Pan et al. (2008) analyzed the AQP1 expression and intratumoral microvessel density (IMD) in endometrioid adenocarcinoma, endometrial hyperplasia, and a normal endometrium. AQP1 was found only in small vessels and microvessels. The AQP1/IMD ratio was significantly higher in endometrioid adenocarcinoma and positively correlated with the histologic grade, invasion, and metastasis [67]. Differently from the AQP1 distribution pattern in endometrial tissue, aquaporin-2 (AQP2) expression is found in the luminal and glandular epithelial cells [79]. Immunohistochemical and Western blot analyses demonstrated a significantly higher expression of AQP2 in EC tissues compared to control samples. In vitro, AQP2 knockdown attenuated migration, invasion, and adhesion but not proliferation in Ishikawa cells [68]. Downregulation of aquaporin-5 (AQP5) showed a reduction in endometrial cancer cells’ migration capacity [69]. Watanabe et al. (2020) associated clinicopathological parameters with AQP3 expression in endometrial cancer samples. Although non-tumoral tissues were not analyzed, the authors demonstrated a significant correlation between AQP3 expression and early tumor stages with lower histological grades [70].

VDAC, also known as a mitochondrial porin, acts as a gatekeeper of mitochondrial metabolites [44]. Jóźwiak et al. (2020) revealed that the isoforms VDAC1 and VDAC3 are upregulated in endometrial cancer tissue compared to a non-tumoral endometrium. Increased expression of VDAC1 was associated with infiltrative endometrial tumors. However, high VDAC3 levels were expressed in poorly differentiated endometrial cancers and low VDAC3 levels in metastatic or advanced tumor stages [71].

## 3. IC Regulation by Steroids Hormones and Growth Factors

Cancer development involves proliferative signaling, resistance to growth suppressors and death, replicative immortality, angiogenesis, and activation of invasion and metastasis pathways [7]. Ion transport mechanisms are implicated in these cell functions by the modulation of ion flux across cell membranes, cell volume, signal transduction pathways, cellular transport [80], and homeostatic maintenance in subcellular organelles [81]. Ion channels’ and transporters’ dysregulation has been related to pathophysiologic processes, especially in epithelial cells [80]. Interestingly, epithelial tissue is the most common site for the development of cancers. Specifically, those epithelia with secretory capacities, such as the uterus, seem to be frequent sites of cancer [82].

The uterus consists of two different layers: the endometrium and myometrium. The endometrium is mainly constituted of endometrial epithelial cells (luminal and glandular cells) underlying stromal cells [83]. In response to monthly reproductive hormone fluctuations and growth factors, endometrial cells possess remarkable plasticity and regenerative capacity to facilitate pregnancy [84]. However, abnormal human endometrium remodeling and regeneration lead to a range of uterine pathologies such as adenomyosis, endometriosis, and endometrial carcinoma [85]. Prolonged exposure to endogenous estrogen effects means an early age at menarche and advanced age at menopause are considered risk factors for EC [86,87]. Although EC mainly affects postmenopausal women, a rare subset of patients is diagnosed during pregnancy [88].

Therefore, various factors are associated with cancer development and progression, such as the modulation of ion channels’ expression through hormones and growth factors [75]. The potassium channels, followed by calcium, sodium, and chloride channels, are the most investigated in several pathologies [89]. The expression of these channels can be modulated by growth factors and hormones, such as the ovarian steroid hormones E2 and P4 [90,91]. There are two isoforms of estrogen receptors (ER): ERα, which predominantly stands out in normal endometrium and early-stage endometrial cancer, and ERβ, which is more evident in late-stage disease and metastasis [92]. P4 functions through two major progesterone receptor (PR) isoforms: PRA and PRB [93].

Since progesterone can suppress the growth of EC cells [94], the expression of PR is inversely related to the clinical grade and stage: lower levels of PR are related to more advanced disease [95]. Endometrial cells treated with P4 increased the expression of CACNA2D3 and the intracellular Ca^+2^ levels, preventing endometrial cancer cell proliferation and inducing apoptosis. In a mouse xenograft model, the treatment with P4 also upregulated the expression of CACNA2D3 and attenuated tumor growth [61].

Hao et al. (2015) identified that 17-β estradiol acts directly in the regulation of calcium Cav1.3 and Cav1.4 channels’ expression. Moreover, 17-β estradiol hormone has been reported to increase Cav1.3 expression in endometrial cancer cells. Furthermore, the decrease in Cav1.3 levels negatively interfered with estrogen-stimulated calcium influx, cell proliferation, and migration of endometrial cancer cells. Therefore, it is suggested that the Cav1.3 channel plays a role in 17-β estradiol-induced carcinogenesis in endometrial cells [59]. According to Bolanz et al. (2008), 17-β estradiol upregulates, in a time-dependent manner, TRPV6 expression in T-47D breast cancer, suggesting that TRPV6 channels facilitate the calcium influx and are part of the molecular mechanism of the 17-β estradiol-induced proliferation in breast cancer cells. [96]. In vitro experiments showed a decline in TRPM4 expression in response to estrogen stimuli in endometrial cancer, possibly involved in cancer cell proliferation and migration [62].

Wang et al. (2018) showed that 17-β estradiol regulated the expression of the KCa1.1 potassium channel in endometrial cancer. Decreased expression of KCa1.1 led to reduced levels of phosphorylated ERK and MEK (p-ERK and p-MEK) proteins. The reduction of KCa1.1 was also related to a decrease in proliferation, migration, and invasion of Ishikawa cells, suggesting that ion channels may be essential regulatory factors to mediate the effects of 17-β estradiol on endometrial cancer cells [56].

Liu et al. (2019) demonstrated that sodium channels provide increased motility, endocytosis, and cell invasion. These channels increase their expression in cancers that are hormone-dependent, such as endometrial cancer, for example [66]. Chlorine channels play a role in cell proliferation, migration, invasion, and metastasis [38]. Studies have suggested that the expression of CLC-3 chloride channels is regulated by 17-β estradiol in breast cancer cells [57,97]. Associations between Na^+^ and Cl^-^ channels have already been described in breast cancer [75]. Zou et al. (2011) demonstrated that AQP2 expression in endometrial Ishikawa cancer cells increased dose-dependently with E2 stimuli; however, AQP2-specific siRNA attenuated E2-enhanced migration, invasion, and adhesion [68].

Ion channels can also be modulated by growth factors such as the vascular endothelial growth factor (VEGF). VEGFs are secreted by fibroblasts and inflammatory cells and bind to their receptors on endothelial cells to promote angiogenesis. However, VEGF receptors’ expression can also be found in tumor cells, resulting in autocrine tumor growth and angiogenesis induction [98]. Several angiogenic factors and their receptors have been studied in a wide variety of tumor types, including breast, pancreatic, lung, prostate, colorectal, brain, and ovarian cancer [99,100,101,102,103,104,105]. Pan et al. (2008) indicated possible signaling cooperation between AQP1 and VEGF to promote angiogenesis in endometrial cancer, facilitating tumor growth and spread [67].

Insulin-like growth factor 1 (IGF1) is associated with a phenotypic change from normal cells to neoplastic cells. There is already an association between IGF1 expression through the action of estrogen in endometrial cancer [106]. Hyperplasic endometrium and endometrial carcinoma tissues express high levels of IGF-I receptor (IGF-IR) [107]. Downregulation of IGF-1R expression inhibits the growth of endometrial carcinoma in vitro [108]. Borowiec et al. (2011) demonstrated that IGF-1 increases the activity and the expression of hEAG channels in breast cancer cells, possibly involved with mitogenic signaling [109]. Furthermore, hERGs can act in mechanisms of tumor metastasis and angiogenesis. K^+^ channels appear to regulate cellular factors involved in cell adhesion signaling, such as β1 integrin, and in increasing basal levels of hypoxia-inducible factor 1α (HIF-1α) and VEGF secretion in the hypoxic tumor microenvironment [49].

## 4. Ion Channels: Biomarkers or Potential Targets for EC?

Tumor-specific expression of certain channel types can form molecular markers of malignancy. By providing a classification for cancer, biomarkers can help to define the clinical prognosis and guide therapeutic strategies [110]. The emergence of new tools such as proteomics allows for identifying molecular fingerprints in EC and serves as a source for clinically relevant biomarkers’ discovery. In addition to assisting in clinical diagnosis and prognostics, proteomics analysis contributes to the evaluation of potential therapeutic targets and mechanisms of therapeutic resistance [111,112]. A prognostic factor has been defined as a patient or disease characteristic/variable that provides an estimation of the recovery or disease relapse chances [113]. EC prognostic factors include the tumoral staging and size, histological cell type determination, and the presence of myometrial and lymphovascular space invasion [114].

Ion channels have also been shown to be involved in endometrial oncogenesis. Tissue analysis revealed different expression patterns of K^+^ [50,54,56], Ca^+2^ [59,61,63], Cl^−^ [65], Na^+^ channels [66], and AQP [67,68,70,71] between endometrial cancer and a nontumoral endometrium. Possibly related to imbalanced hormonal signaling, the increased ion channel expression appears to be linked to the channel-mediated pathway required for endometrial tumor progression.

The different expression patterns of ion channels between tumor and non-tumor tissues/cells also highlighted the ion channels that may make potential targets for anticancer therapies [115]. Based on preclinical in vitro and in vivo studies, channel inhibitors or channel downregulation may suppress endometrial cancer cell proliferation, differentiation, migration, and invasion, leading to tumor growth suppression [51,54,55,56,57,58,59,60,62,64,65,66,68,69]. CACNA2D3 calcium channel expression showed the opposite effect to the other channels in endometrial cancer. Its downregulation showed involvement in proliferation, migration, and tumor growth [61]. Considering that ion channels are widely expressed in the tissues and have physiological importance for the body’s homeostasis regulation, these data highlighted the complexity and importance of tracking the expression patterns of ion channels according to the type of tumor under analysis.

However, ion channels as a therapeutic target could bring side effects and risks since many of the ion channels identified in cancer cells are expressed in healthy normal cells [115]. Yet, theoretically, a plausible treatment for cancer regarding the functions of ion channels should target those mechanisms involved in tumor progression, such as proliferation, migration, and invasion. Moreover, they are easily accessible because they are membrane proteins that are often overexpressed or activated in cancer [116]. In this way, TRP channels, Cav1.3, KCa3.1, and AQP2 are all candidates to be targeted by therapies. Indeed, several in vivo studies evaluated the systemic effect of ion channels as a pharmacological target. In general, Ca^+2^, K^+^, and Na^+^ channels’ inhibition or activation demonstrated a lack of specificity and side effects mainly on the cardiovascular system [117]. For example, although the use of hERG channel blockers triggers cell cycle arrest and apoptosis in cancer cell lines [49], hERG1 channels are essential for regulating the cardiac action potential. Inherited mutations or pharmacological blocks that cause loss of channel function can lead to life-threatening arrhythmias. These proarrhythmic side effects require significant attention in new cancer drug development [118].

Although ion channel-targeting strategies may have off-target effects, some early-phase clinical trials are under study for cancer treatment [115]. TM-601 is a synthetic version of peptide chlorotoxin, found in scorpion venom, which acts as a Cl^-^ channel activity blocker. Intracavitary administration of TM-601 radiolabeled with Iodine-131 in patients with recurrent glioma (phase 1 clinical trial) demonstrated good tolerability and potential antitumoral effects [119]. Another therapeutic approach is the use of monoclonal antibodies targeting ion channels [120]. P2X7 is a transmembrane receptor expressed in various cell types that can form a nonselective channel for cations when activated by extracellular ATP [121]. A non-functional isoform of P2X7 (nfP2X7) appears significantly expressed in tumor cells, such as those of bladder, kidney, colorectal, and lung cancer [122]. The use of a monoclonal antibody targeting an epitope on the cancer-specific variant of nfP2X7 (phase 1 clinical trial) was well-tolerated and brought promising results in basal cell carcinoma treatment [123]. The use of SOR-C13, a TRPV6 calcium channel inhibitor, in patients with advanced solid tumors (phase 1 clinical trial), including ovarian, colorectal, non-small cell lung, and pancreatic, demonstrated disease stabilization and suggested potential antitumor activity [124].

Approved drug repurposing is another field to be explored in the targeting of ion channels for cancer therapy. Drugs currently used in hypertension and psychiatric disorders’ treatment, for example, have inhibitory effects on ion channels, and their redirection may be promising for cancer treatment [125]. For example, the imbalance in intracellular calcium levels’ homeostasis related to estrogen signaling in carcinogenesis highlights the promising use of calcium channel blockers in endometrial cancer treatment [126]. It is noteworthy that drug repurposing should include new drug delivery and formulation methods since the expected effects for cancer treatment require higher doses than those used for other conditions [126].

## 5. Conclusions

**Figure 1:**
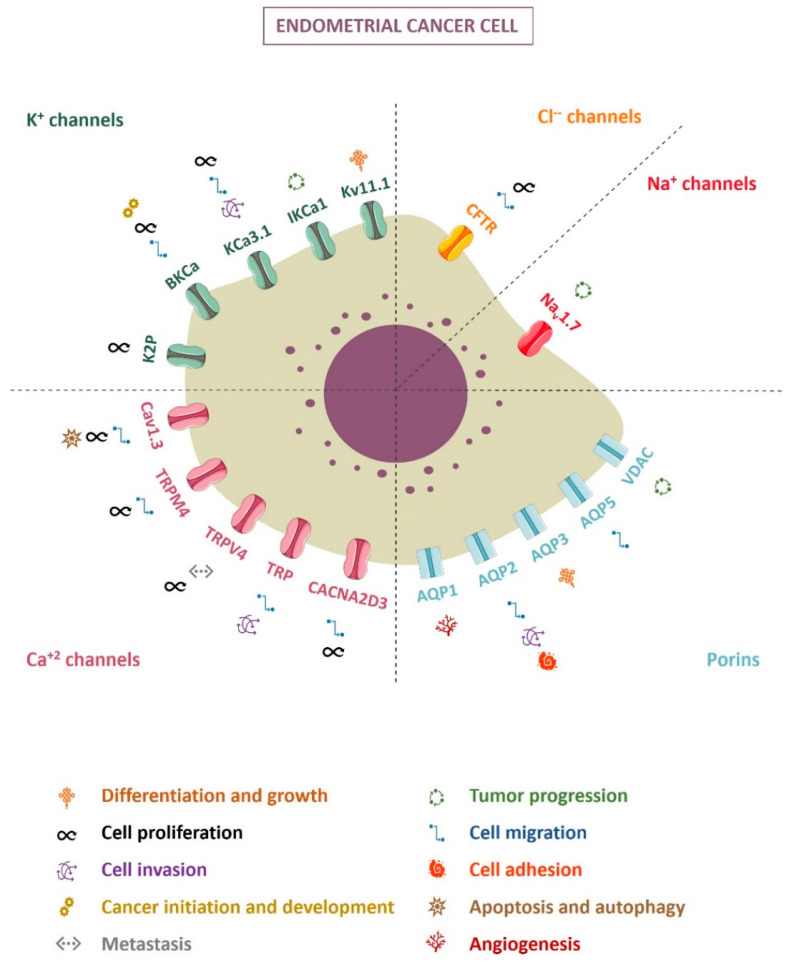
Ion channels and their involvement in endometrial cancer cells.

More than regulators of the flow, ion channels appear to be the leading figure in a myriad of processes, including carcinogenesis. Figure 1 summarizes the ion channels with a described role in endometrial cancer. Today, the ion channel research grand challenge consists of determining and selectively blocking ion channel subtypes or ion channel mutants according to the tumor type, along with searching for safer pharmacotherapy [127]. Although there are not any clinical trials to date that validate the use of ion channels as molecular markers or therapeutic targets in endometrial cancer, the data presented highlight the role of ion channels in endometrial tumor progression, with a promising therapeutic approach to be investigated.
